# Fasting-dependent Vascular Permeability Enhancement in Brown Adipose Tissues Evidenced by Using Carbon Nanotubes as Fluorescent Probes

**DOI:** 10.1038/s41598-018-32758-8

**Published:** 2018-09-27

**Authors:** Masako Yudasaka, Yohei Yomogida, Minfang Zhang, Masako Nakahara, Norihiko Kobayashi, Takeshi Tanaka, Yuko Okamatsu-Ogura, Kumiko Saeki, Hiromichi Kataura

**Affiliations:** 1 Nanomaterials Research Institute, National Institute of Advanced Industrial Science and Technology, Tsukuba, Ibaraki, 305-8565 Japan; 2 Graduate School of Science and Technology, Meijo University, Nagoya, 468-85002 Japan; 3 CNT-Application Research Center, National Institute of Advanced Industrial Science and Technology, Tsukuba, Ibaraki, 305-8565 Japan; 4 Department of Disease Control, Research Institute, National Center for Global Health and Medicine, Shinjuku-ku, Tokyo, 162-8655 Japan; 5 Department of Biomedical Sciences, Graduate School of Veterinary Medicine, Hokkaido University, Sapporo, 060-0818 Japan

## Abstract

Brown adipose tissue (BAT), which is composed of thermogenic brown adipocytes (BA) and non-parenchymal components including vasculatures and extracellular matrix, contribute to the maintenance of body temperature. BAT distribution is detected by positron emission tomography-computed tomography (PET/CT) using ^18^F-fluorodeoxy glucose (^18^F-FDG) or single-photon-emission computed tomography-computed tomography (SPECT/CT) using [^123^/^125^I]-beta-methyl-p-iodophenyl-pentadecanoic acid. Although sympathetic nerve activity and thermogenic capacity of BA is downregulated under fasting conditions in mice, fasting-dependent structural changes and fluid kinetics of BAT remain unknown. Here we show that the fasting induces fine and reversible structural changes in the non-parenchymal region in murine BAT with widened intercellular spaces and deformed collagen bands as revealed by electron microscopy. Interestingly, a newly introduced near infrared fluorescent probe of single-walled carbon nanotubes (CNTs) coated with phospholipid polyethylene glycol (PLPEG) easily demonstrated enhanced vascular permeability in BAT by the fasting. PLPEG-CNTs extravasated and remained in intercellular spaces or further redistributed in parenchymal cells in fasted mice, which is a previously unknown phenomenon. Thus, PLPEG-CNTs provide a powerful tool to trace fluid kinetics in sub-tissue levels.

## Introduction

Brown adipose tissue (BAT) exerts anti-obesity and anti-diabetes effects, and therefore, is attracting attention as a new target of the drug discovery for the treatment of metabolic syndrome. Currently, ^18^F-fluorodeoxyglucose-based positron-emission tomography coupled with computed tomography (^18^F-FDG-PET/CT) is the only measure to visualize BAT in humans. In ^18^F-FDG-PET/CT, BAT signals are enhanced by cold stimulations. Although cold stimuli up-regulate the activity of sympathetic nerves, which is the major regulator of BAT activity in mice, sympathomimetics fail to enhance ^18^F-FDG signals in human BATs^1^. In addition, human BAT signal in ^18^F-FDG-PET/CT is increased by fasting^2^ while the activity of BAT-governing sympathetic nerves is lowered under fasting conditions in mice^3^. Despite these discrepant findings, human and murine BATs share substantial similarities in gene expression profiles^4^ and distributions in the body^5^. Brown adipocytes (BA) utilize lipids as the primary energy source, whereas signals in ^18^F-FDG-PET/CT reflect their glucose-uptake activities, but not the rate of their energy expenditure *per se*. Therefore, signal intensities in ^18^F-FDG-PET/CT in BAT would not always reflect their thermogenic capacities and certain fine morphological changes might possibly take place to enhance glucose accumulations in BAT independently of the level of sympathetic nerve activity.

In adipose tissues in general, fasting induces shrinkage of lipid droplets reflecting enhanced lipolysis to supply materials for gluconeogenesis. When we examined the histology of BAT specimens in starved mice, we determined not only the reduction of lipid droplet sizes and numbers but also alteration in the structure of intercellular spaces. Although altered interstitial structures may reflect the change in the fluid kinetics in the sub-tissue level, it takes considerable time and labor to find out such fine morphological changes by histological examinations. Moreover, real-time tracing of fine structural changes cannot be carried out by histological observations using fixed specimens.

A new probe of single-walled carbon nanotubes (CNTs)^6,7^ is known to emit fluorescence in the near infrared (NIR) region around 1000–1400 nm^8^, and works as an excellent probe when coated with phospholipid polyethylene glycol (PLPEG-CNT) for vascular imaging by following reasons. Light in this region can penetrate deeply into the body due to its low absorption and is weakly scattered by biomolecules. In addition, biomolecule-derived auto-fluorescence level is low in this wavelength region. Accordingly, CNT NIR-fluorescence-based bio-imaging techniques have an advantage that they show high signal-to-noise ratios^9–12^. Due to the high bio-affinity property of PLPEG and the small size of CNT, PLPEG-CNT shows very low binding affinities to biomolecules^13^, which is suitable to image the vascular systems^14–17^ and, further expected to find the fluid kinetics in sub-tissue levels.

Therefore, PLPEG-CNT was administrated into starved mice to trace the blood flow, and possibly, interstitial fluid in BAT. Surprisingly, intravenously administrated PLPEG-CNT fluorescent probe revealed that the vasculature permeability in BAT was specifically enhanced by the fasting. This unexpected finding presents a potential of PLPEG-CNT as a new tool to examine fluid kinetics in sub-tissue levels.

## Results

### Fasting-induced accumulation of PLPEG-CNTs in BATs coupled with specific morphological changes

**Figure 1:**
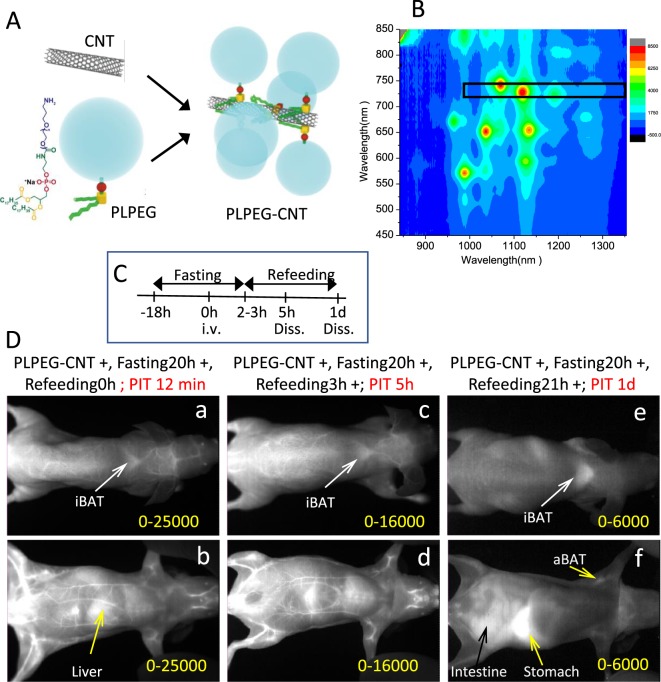
(**A**) Schematic illustration of PLPEG, CNT, and a supposed structure of PLPEG-CNT. (**B**) Fluorescent spectrum of PLPEG-CNT dispersed in water. The black rectangular in “B” denotes the excitation and fluorescence wavelength region used in the imaging. (**C**) Time table of fasting, feeding, intravenous injection (i.v.) of PLPEG-CNT, and dissection (Diss.). (**D**) Images of fasted mice at PIT 12 minutes (a,b), 5 h (c,d), and (1D,e,f). Dorsal images (a,c,e) and ventral images (b,d,f). Image-signal-intensity ranges denoted with yellow numbers are optimized for the whole-body visualization at each time point (n = 4).

In our previous study, we showed that CNT coated with a phosphoryl choline-based compound, poly (2-methacryloyloxyethyl phosphoryl choline-co-n-butyl methacrylate, selectively accumulated in the capillary endothelial cells in adipose tissues of mice within several hours after intravenous injection^18^. To further evaluate the impact of the character of the coated materials, we re-performed whole-body imaging using CNT coated with phospholipid-based compound of PLPEG (Fig. 1A), in relation with feeding conditions (Fig. 1C). The CNT length ranged from micrometers to tens nanometers, and its diameter was about 1 nm. Its diameter after the PLPEG coating would be about 14 nm as estimated from the occupied area estimation of PLPEG that adsorbed on the nanocarbons (Fig. 1A)^19^. The wavelength region employed for the fluorescence imaging is indicated with a black rectangular in the fluorescence spectra (Fig. 1B)^17,18^. The fluorescence spectrum presented sharp peaks characteristic of well-dispersed CNTs^8^.

Mice were kept under either fasting for 18 hours or ad lib feeding conditions before the administration of PLPEG-CNT (Fig. 1C). Consistent with previous reports^14–17^, PLPEG-CNT successfully imaged systemic vasculature networks at post injection time (PIT) 12 minutes (Fig. 1Da,b) in fasted mice. The vasculature images faded away with time, being still visible at PIT 5 h (Fig. 1Dc,d), and then undetectable at PIT 1d (Fig. 1De,f). Surprisingly, BATs of fasted mice, which were imaged as early as at PIT 12 minutes (Fig. 1Da,b) or 5 h (Fig. 1Dc,d), showed even clearer images at PIT 1d, when the vasculature system had already become undetectable (Fig. 1De,f).

Here, the gastrointestinal system created bright images at PIT 5 h and 1d due to auto-fluorescence of refeeding-food ingredients (Fig. 1Dd,f), which was similar for mice with no PLPEG-CNT injection (Supporting Data 1).

**Figure 2:**
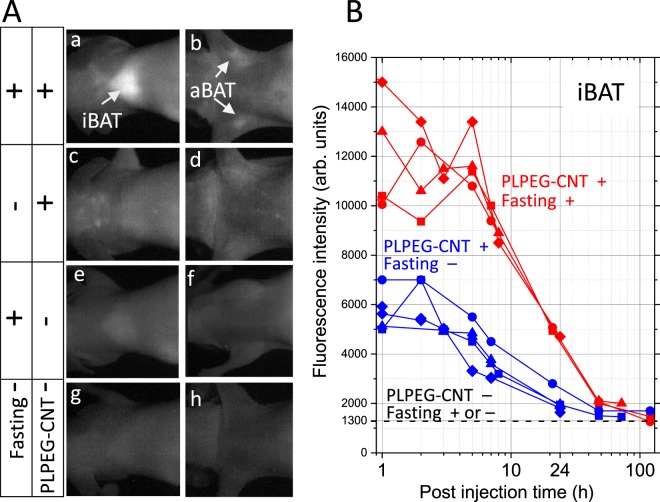
**(A)** Fasting-induced BAT imaging with PLPEG-CNT. NIR Images of iBATs and aBATs with their arounds at post injection time of 1d. CNT accumulating area is brightly imaged. The brightness and contrast levels of all the images in (A) are the same. **(B)** Time course of PL intensities at iBAT for (PLPEG-CNT+, Fasting+) and (PLPEG-CNT+, Fasting−) conditions. The PL intensities denote the averaged intensities at the brightest area. The PL intensities at iBATs for “PLPEG-CNT−, Fasting− ” mice were about 1300 in (**B**). iBAT: interscapulum BAT, aBAT: axillary BAT (n = 4).

The bright BAT images were fasting-dependent: The fasted mice showed brighter interscapular BAT (iBAT) and axillary BAT (aBAT) (Fig. 2Aa,b) than non-fasted (ad lib feeding) mice (Fig. 2Ac,d). Time course studies indicated that the iBAT of fasted mice was always brighter than that of non-fasted counterparts (Fig. 2B). Without PLPEG-CNT administration, BATs remained dark regardless of whether mice were kept in fasted states (Fig. 2Ae,f) or not (Fig. 2Ag,h).

**Figure 3:**
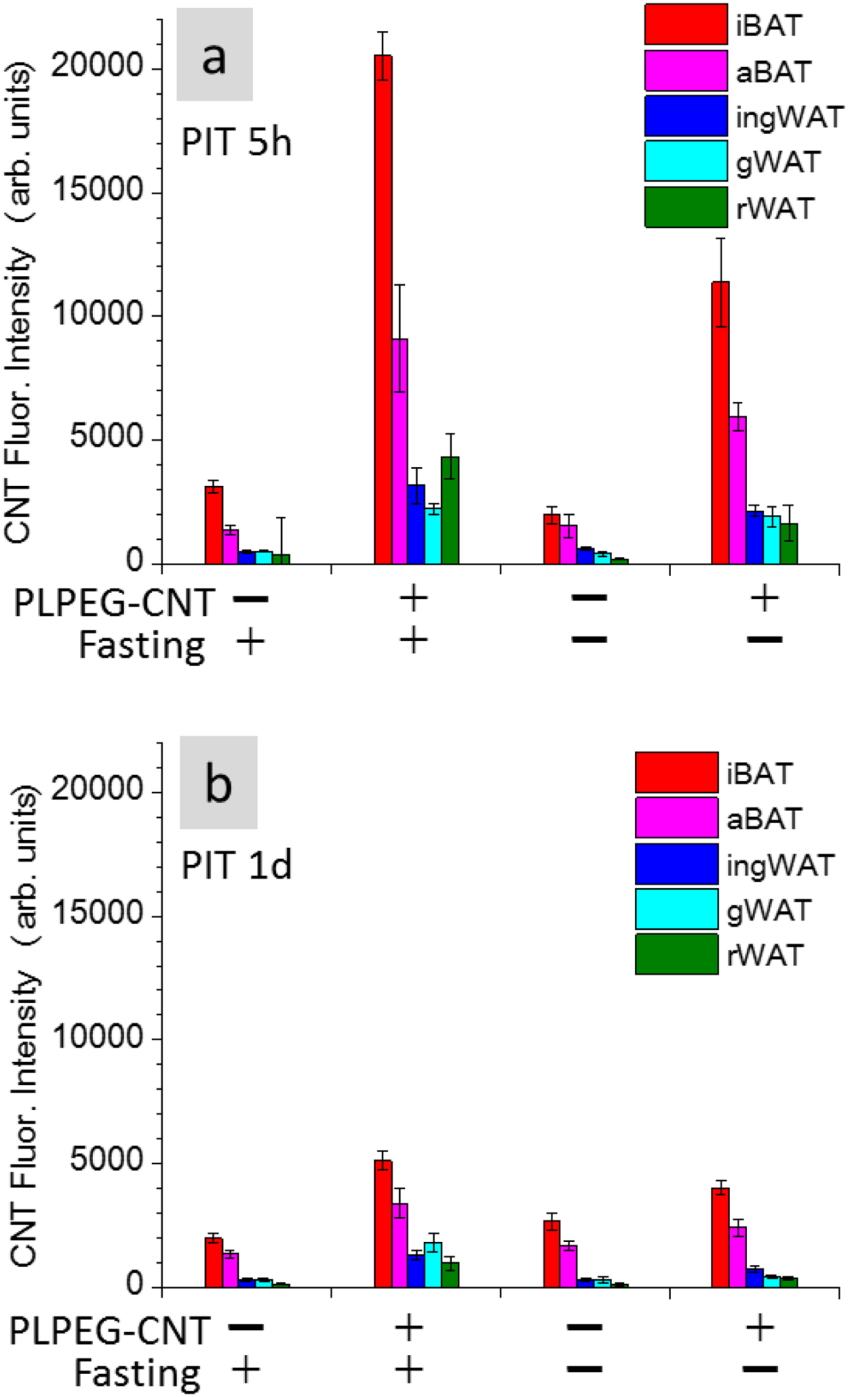
Fluorescence intensities of iBAT, ingWAT, aBAT, gWAT, and rWAT embedded in paraffin blocks. The tissues were taken at PIT 5 h (**a**) and 1d (**b**). Back ground fluorescence intensity of 1500 was subtracted. iBAT: interscapulum brown adipose tissue, aBAT: axillary brown adipose tissue, ingWAT: inguinal white adipose tissue, gWAT: gonadal white adipose tissue, rWAT: retroperitoneal white adipose tissue (n = 5).

The fasting-depending up-regulation of the signal intensity of BAT was further confirmed by measuring the CNT-NIR-fluorescence intensity of the tissues dissected at PIT 5 h and PIT 1d. We determined that BATs of the fasted mice were significantly brighter than those of non-fasted mice (Fig. 3).

**Figure 4:**
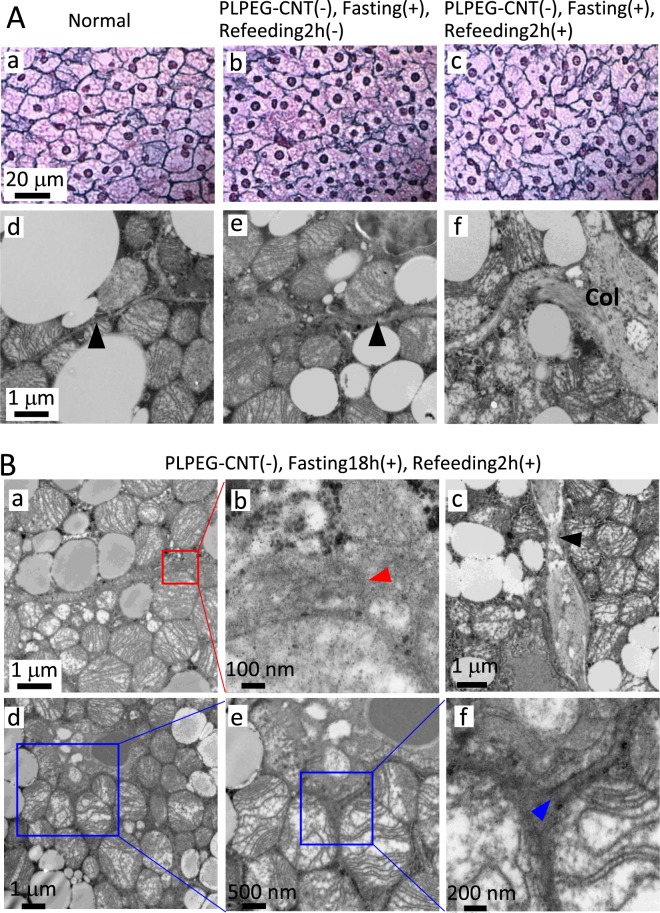
**(A)** Optical micrographs of iBATs stained with silver-impregnation (a–c), and TEM images of iBAT stained with Os (d–f) for a normal mouse (PLPEG-CNT−, no fasting) (a,d), a mouse (PLPEG-CNT−, fasting18h+, Refeeding2h−) (b,e), and a mouse (PLPEG-CNT−, fasting18h+, Refeeding2h+) (c,f). Reticular fibers (type III collagen) was stained black with silver impregnation. Black arrow heads point the inter-cellular spaces. (n = 2) **(B)** TEM of iBAT of a mouse (PLPEG-CNT−, fasting18h+, Refeeding2h+). An end of the collagen band (a,b, red arrow head), narrow part of collagen (c, black arrow head), and the gap junction (d–f), showing its end (f, blue arrow head) (n = 1).

From these findings, we hypothesized that iBAT-selective imaging for the fasted mice was attributed to certain histological alterations, which induced the prolonged retention of PLPEG-CNT in iBAT. Morphological examinations revealed that the structure of reticular fiber (type III collagen) was disarranged (Fig. 4Aa,b) and the intercellular spaces were widened (Fig. 4Ad,e) in fasted mice. These changes persisted at least during the successive 2h-refeeding (Fig. 4Ac,f). The widened intercellular spaces in iBATs of the fasted and fasted-refed mice were confirmed by transmission electron microscopy (TEM) (Fig. 4Ag–i). The ends of broken collagen bands (Fig. 4Ba,b, red arrow head) along with thinned collagen bands (Fig. 4Bc, black arrow head) were detected. Moreover, gap junctions showed aberrant structures, some of which were separated by approximately 200 nm (Fig. 4Bf, blue arrow head) in contrast to 20 nm for the normal gap junction as observed in the iBAT of ad lib fed mice (Supporting Data 2).

Collectively, fasting induced unique and distinctive fine morphological changes in iBAT especially in its interstitial regions.

The fasting-induced structural changes in BAT were significantly alleviated at PIT 1d as inferred from the histological observation (Supporting Data 3), suggesting that the tissue structure changes caused by the fasting would be a reversible process.

**Figure 5:**
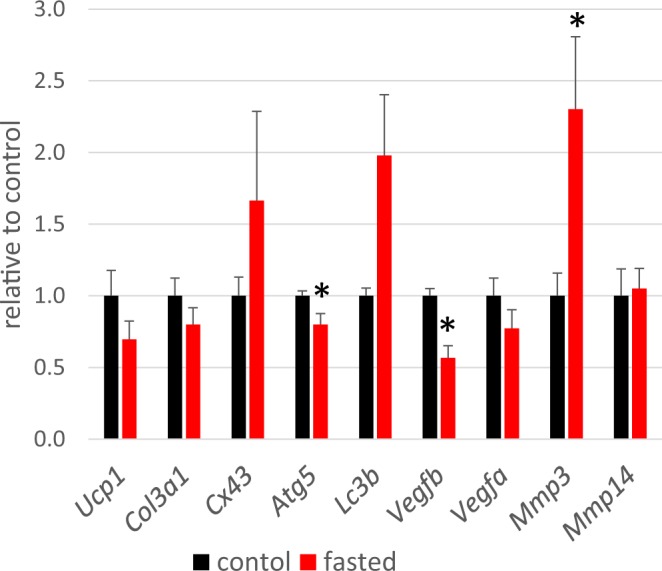
Gene expression in iBATs of control mice (PLPEG-CNT−, fasting 0 h, refeeding 0 h) (black) and fasted mice (PLPEG-CNT−, fasting 20 h, refeeding 0 h) (red). Data were normalized to the expression of ActB and expressed as relative value to the control. The black asterisks denote the red bar values are significantly different from the black bar values (n = 5).

To obtain mechanistic insight into the fasting-dependent morphological changes of iBAT, gene expression profiles were examined. It was found that the mRNA expression level of matrix metalloproteinase (MMP) 3, which is known to digest collagen type III, was significantly increased by the 20 hours-fasting, while *Mmp1* mRNA was undetected and *Mmp14* expression level was not affected by the fasting (Fig. 5). These data suggest that the degradation of the type III collagen of reticular fibers were induced by fasting and may contribute to the specific morphological change of the collagen bands. The mRNA expression levels of *Cx43* and or Type 3 collagen (*Col31*) were not reduced by the fasting, suggesting that the disorders of the gap junction and collagen bands were not resulted from downregulated synthesis of these proteins. Autophagic degradation of Cx43^20,21^ does not seems to be mainly involved in the induction of iBAT structure disorders either, because the expressions of autophagy-related genes (*Atg5*) was not increased by fasting (Fig. 5) or we did not detect any autophagosomes in iBAT of fasted mice (data not shown).

### Fasting-induced changes in iBAT fluid kinetics as evidenced by PLPEG-CNT histological observation

To examine whether there were any changes in the fluid flow conditions in iBAT, the histology of iBAT of PLPEG-CNT-administrated mice were examined by NIR fluorescence microscopy.

**Figure 6:**
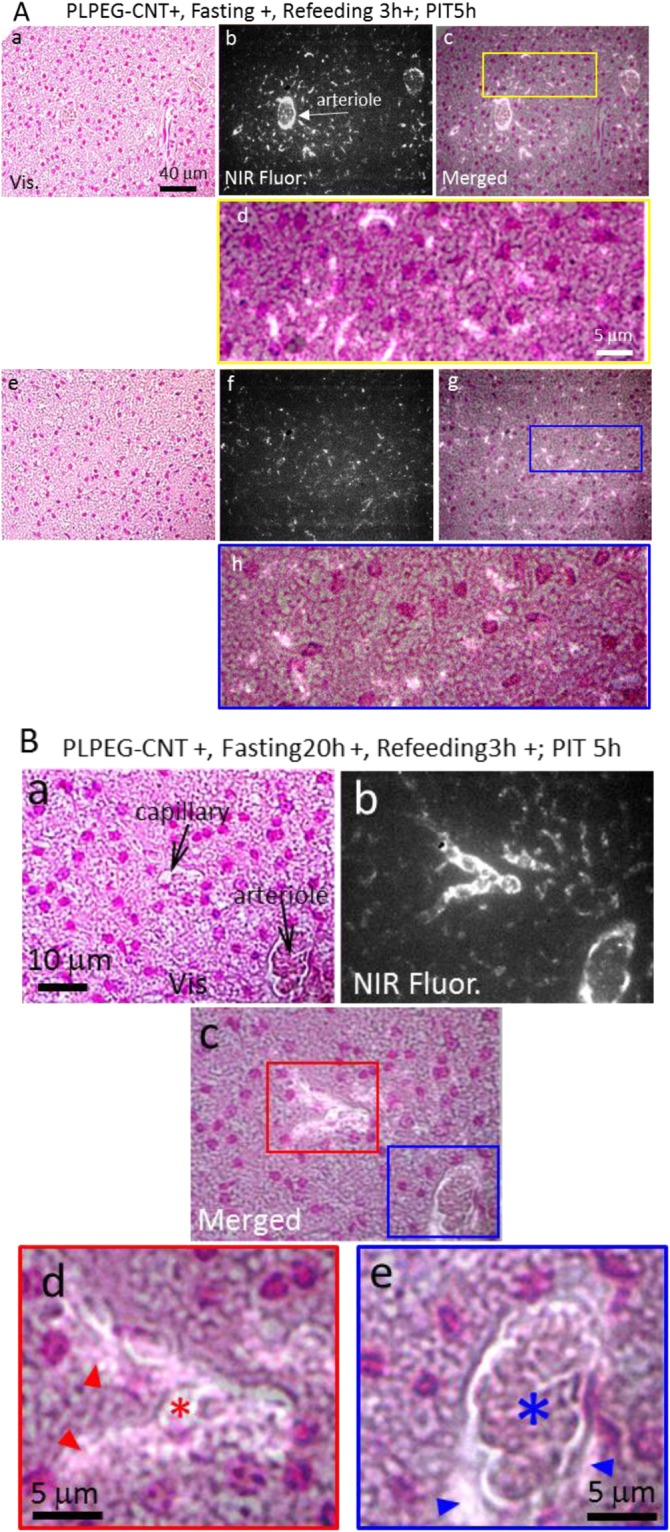
**(A)** Micrographs of iBATs of mice (PLPEG-CNT+, Fasting20h+, Refeeding 3 h+) at PIT 5 h. Micrographs taken near arteriole (A,a–d) and apart from the arteriole (A,e–h). **(B)** Higher magnification micrographs of the iBAT near the vessels. CNT accumulating area is brightly imaged. Red and blue arrowheads denote the PLPEG-CNTs existing in pericapillary and periarteriolar spaces, respectively. Red and blue asterisks denote the capillary and arteriole, respectively. The iBAT tissue slices were stained with nuclear fast red, whose fluorescence in the NIR region was weak enough to identify the NIR fluorescence of CNTs. Vis: visible-light transmission micrograph. NIR Fluor.: NIR fluorescence micrograph. Merged: merged image of vis and NIR Fluor micrographs (n = 5).

Coinciding with the iBATs imaging results shown in Fig. 2, the iBAT of the fasted mice showed higher numbers of CNT-fluorescent spots than that of the non-fasted mice (ad lib feeding) in the histological observation (Supporting Data 4). In the fasted mice, the PLPEG-CNTs distributed more densely adjacent to arterioles (Fig. 6Aa–d) than in the other areas (Fig. 6Ae–h). In the higher magnification images (Fig. 6Ad,h), the CNT-fluorescent spots were detected in perinuclear regions of BAs as well as intercellular spaces of iBAT. Similar patterns of PLPEG-CNT distribution were observed for aBAT (Supporting Data 5).

The arteriole and its proximate areas in iBAT were further examined at higher magnification (Fig. 6B). In addition to the dense circumferential CNT-fluorescent signals at the inner surface of arteriolar walls (Fig. 6Be, blue asterisk), spotted CNT-fluorescent signals were detected in the periarteriolar space (Fig. 6Be, blue arrow heads). Similarly, bright CNT-fluorescent spots were detected in the pericapillary space (Fig. 6Bd, red arrow heads) in addition to the capillary lumen (Fig. 6Bd, red asterisk). These results indicate that the extravasation of PLEG-CNTs was induced by the fasting in iBAT, and PLPEG-CNTs diffused into the intercellular spaces and subsequently entered the BAs.

### Ultrastructural localization of extravasation of PLEG-CNTs

Extravasation of PLPEG-CNTs from the arteriole and the capillary in iBAT suggested by the optical microscopic observation results (Fig. 6) were further supported by the findings in TEM.

**Figure 7:**
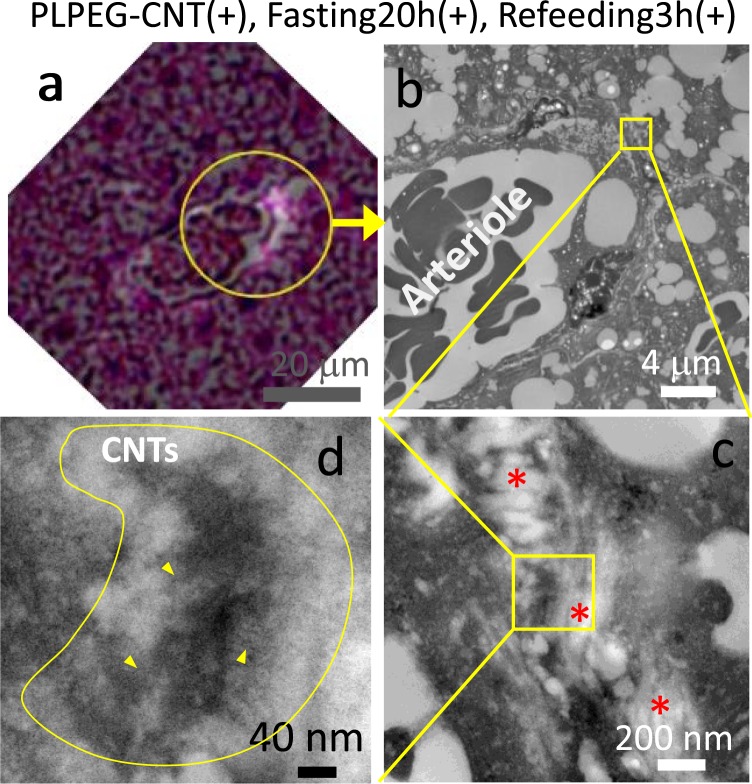
Micrographs of iBAT of the mouse (PLPEG-CNT+, fasting20h+, Refeeding3h+; PIT5h). A merged micrograph of a visible-light image and NIR-fluorescent image of a slice taken from the same paraffin block used for “Fig. 6B” (**a**). TEM images of an area in the circle of “a” (**b–d**). Tissue thickness of “a” and “b-d” were 5 μm and 80 nm, respectively. The CNT-containing agglomerate is highlighted by enclosing with a yellow line in “d.” Yellow arrow heads point some of the CNTs among many others. Red asterisks in “c” denote collagens (n = 1).

*In vitro* observations, PLPEG-CNTs showed the appearance of fibers, which corresponds to the individual CNTs with diameters of about 1 nm, or-bundle-like appearances (Supporting Data 6). For *in vivo* observations, the tissue slice (5 μm thick) used for optical microscopic observations (Fig. 7a) was separated from the glass plate and treated it for TEM (Os staining, 80 nm thick). The encircled area in Fig. 7a corresponds to the TEM images in Fig. 7b-d. In TEM images (Fig. 7b–d), a dark agglomerate (Fig. 7d, yellow line area) was detected in the periarteriolar space near collagen bands (Fig. 7b,c) just at the region corresponding to the area with bright signals in near-infrared photoluminescence microscopy (Fig. 7a). From its morphology, it appeared to be the agglomerate of fibrous materials. Observing this object closely, a lot of fibrous materials with hundreds to tens nm were visible, for example, as pointed with yellow arrow heads in Fig. 7d. Each of them was most likely to be a part of bending/curled CNT existing at the focal point, strongly suggesting that the dark agglomerate contained CNTs. It was not easy to detect individual CNT particles in the thick CNT agglomerates by TEM because the thick CNT agglomerates as well as the biomaterials constructing the tissues hinder the clear imaging of individual CNTs.

### PLPEG-CNTs moved to lymph nodes

Since PLPEG-CNTs show low binding affinities to biomolecules in general^13^, the interaction between PLPEG-CNTs and connective tissues in inter-cellular spaces should be weak, and thus, considerable amounts of PLPEG-CNTs would promptly drained from the fluid of parenchyma to the lymphatic vessels.

**Figure 8:**
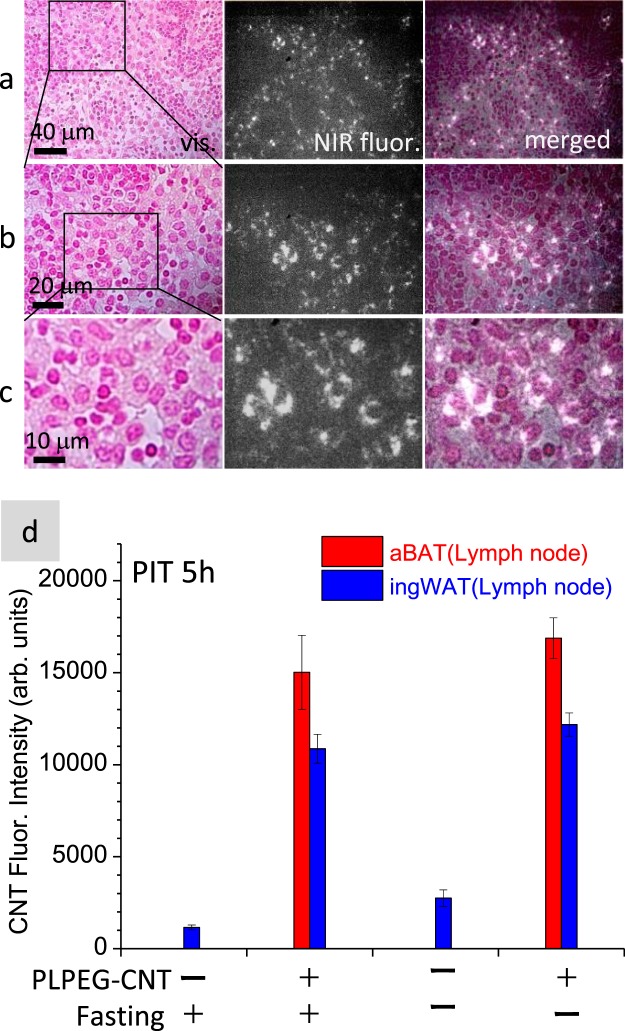
NIR fluorescence micrographs of aBAT-LNs of a mouse (PLPEG-CNT+, Fasting20h+, Refeeding3h+; PIT 5 h) observed under different magnification (**a–c**). CNT accumulating areas are brightly imaged. Fluorescence intensities of lymph nodes in aBAT and ingWAT embedded in paraffin blocks (**d**). The tissues were taken at PIT 5 h. Back ground fluorescence intensity of 1500 was subtracted (n = 5).

As expected, remarkable amounts of PLPEG-CNTs were found in the draining lymph nodes (LNs) of aBAT (aBAT-LNs) (Fig. 8, Supporting Data 7), ingWAT-LNs (Supporting Data 8), and submandibular LNs (Supporting Data 9) irrespective of feeding conditions for PIT 5 h and PIT 1d. Figure 8a shows micrographs of aBAT-LNs of the fasted mice at PIT 5 h, where many CNT-fluorescence spots were localized in medullary cords and medullary sinus. In the medulla of LNs, CNTs bright spots often existed in the perinuclear regions of reticular cells, but not in macrophages (Fig. 8b,c). There was no significant difference in the CNT fluorescence intensities in aBATs-LNs or ingWAT-LNs at PIT 5 h between fasted and non-fasted mice (Fig. 8d). It is known that there are large LN complexes in the axillary, inguinal and submandibular regions, collecting miscellaneous foreign substances administrated in a living body including PLPEG-CNTs. Therefore, it is not surprising that PLPEG-CNTs, which were initially distributed to broader regions in the body, were promptly relocated to those major LN complexes independently of feeding conditions.

## Discussion

### Extravasation of PLPEG-CNTs in BAT

**Figure 9:**
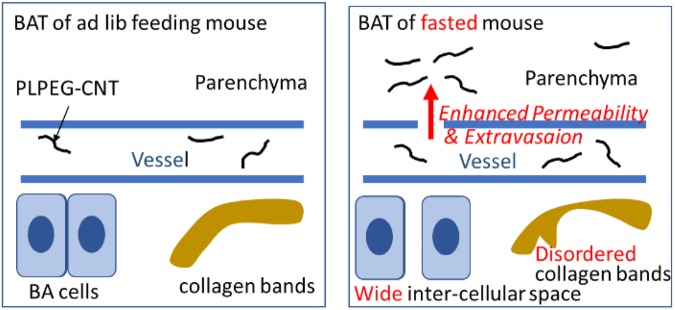
Schematic explanation to present the effect of fasting on BAT structure and fluid kinetics.

Fasting enhanced extravasation of fluid through degenerated basal membranes of capillary/arteriolar walls as demonstrated by disarranged or broken collagen bands in the extracellular matrix in BAT of fasted mice (Figs 6–7), which is schematically presented in Fig. 9.

The widening of inter-cellular spaces and disorders of collagen bands may be attributed, at least in part, to the fasting-induced proteolytic events, as confirmed by the increase of Mmp3 mRNA levels. According to the gene expression data that are stored in BioGPS database^22^ using dataset of GeneAtlas MOE430, gcrma^23^, Mmp3 expressions in BAT (259.57 ± 120.44) are significantly higher than 3 x median value (3 × 6.3 = 18.9). Indeed, BAT is the 8^th^-highest Mmp3 mRNA-expressing tissue. Interestingly, lymph node (794.36 ± 447.47) is the 4^th^-highest tissue. The reason why Mmp3 expression was upregulated in iBAT by fasting is unknown, which remains to be elucidated by future studies.

The extracellular matrix disorders induced by the fasting could cause the enhancement of the extravasation in BAT. The vascular permeability enhancement is often related to the VEGF pathway^24^, which is not the case in our study because the mRNA expression levels of *Vegfa* and *Vegfb* did not increase by the fasting (Fig. 5).

Although the reason why acute fasting induces permeability only in BAT remains elusive, it may possibly be related to unique characters of vascular endothelial cells in BAT (BAT-EC). The BAT-ECs are highly unique in several points. Firstly, they serve as a progenitor of brown adipocyte (BA) in adult mice^25,26^ in contrast to the fact that fetal BA is originated from myoblast^27^. Secondly, BAT is an active remodeling tissue coupled with highly dynamic behaviors of BAT-ECs, which commence massive proliferation under cold environment^28^. Although we have not yet determined whether or not fasting enhances the activity of BAT-ECs, particularly high proliferation/motility capacities of BAT-ECs, which may cause up-regulated permeability of BAT vasculatures, guarantee active remodeling potential of BAT to promptly respond to altered metabolic states during fasting, cold acclimation and so on.

The accumulation of PLPEG-CNTs in iBAT of the fasted mice re-increased when the refeeding started (cf. PIT 5 h in Fig. 2). This mechanism is unclear. It might be due to the leptin amount increase induced by the re-feeding after the fasting^29^, and leptin increases adipose tissues capillary permeability^30^, which needs further confirmation.

Although the accumulation of PLPEG-CNT in WATs is rather low, they also showed similar fasting-dependent enhancement of NIR fluorescent signals (Fig. 3). Therefore, analogous morphological changes as a result of enhanced extravasation of PLPEG-CNT might also be taken place in WAT of fasted mice although histological studies did not show clear evidence because of severe shrinkage of WAT in starved mice. Interestingly, the areas where PLPEG-CNT distributed corresponded to the regions where beige-like cells located (Supporting Data 11). This finding suggests that the fasting-induced extravasation occurred more frequently in beige-cell rich area in WAT of staved mice, which also needs further confirmation in future.

### Plasma protein transfer to PLPEG-CNT

In contrast to the case of CNTs coated with an amphiphilic and biocompatible polymer, poly(2-methacryloyloxyethyl phosphorylcholine-co-n-butyl methacrylate) (PMB-CNT), which specifically accumulates in the capillary endothelial cells in adipose tissues^18^, PLPEG-CNTs were localized in peri-capillary/peri-arteriolar areas as well as parenchyma and intercellular spaces in iBAT. Accumulation of PMB-CNTs in adipose tissue capillary endothelial cells is due to the presence of apolipoproteins on their surfaces *via* transfer from plasma lipoproteins^18^. By contrast, the transfer efficiency of apolipoproteins onto PLPEG-CNTs was as low as that of albumin (Supporting Data 12), which is reported to be scarcely absorbed by PLPEG-CNTs^13^. Thus, it is reasonable that the PLPEG-CNTs were not detected in the capillary endothelial cells in adipose tissues including BAT.

### PLPEG-CNTs in hepatic stellate cells

Besides BATs and lymph nodes, PLPEG-CNTs accumulated in the hepatic stellate cells (Supporting Data 13–15). As we previously reported, PMB-CNT was ingested by hepatic stellate cells probably by apolipoproteins transferred to PMB-CNT in plasma^18^. PLPEG-CNTs, however, hardly absorb apolipoproteins (Supporting Data 12). It may be possible that hepatic stellate cells ingested PLPEG-CNTs and PMB-CNTs simply by a physical reason since they are nanometer-sized small particles with fibrous structures. The precise mechanism of PLPEG-CNTs accumulation in hepatic stellate cells will be the subject of future research.

### Comparison with ^18^F-FDG-PET/CT

Currently, the BAT imaging is generally performed by ^18^F-FDG-PET/CT. Glucose is up-taken via transporter proteins existing on the plasma membrane of BA. By preconditioning with cold stimulations (19 °C, 2 hr)^31^ glucose uptake by BA is significantly enhanced due to upregulated activity of the sympathetic nerve, which activates AMP-dependent protein kinase (AMPK), thus enhancing membrane translocation of Glut4 protein to augment glucose uptake by BA. Since BAT are intensively innervated by the sympathetic nerve system, ^18^F-FDG-PET/CT provides the “BAT over WAT imaging”. Not only by acute cold stimulations, cold acclimation enhances the activity of BAT to up-regulate its thermogenic activity for an adaptation to cold environments^32–35^. Moreover, the thermogenic activity of BAT is even enhanced under postprandial conditions to expend a calorie surplus toward the optimal energy balance in a living body^36,37^. On the other hand, BAT activity is repressed under fasted conditions by the decline of sympathetic nerve activity in mice, contributing to saving energy expenditures by suppressing thermogenesis-mediated energy loss^38^. The discrepancy between enhanced signal intensities in ^18^F-FDG-PET/CT in humans and lowered thermogenesis activities of BAT in mice under fasted conditions remains unsolved. Our finding that the vascular permeability in BAT is enhanced in fasted mice suggests that the fasting-depending ^18^F-FDG-PET/CT signal enhancement in humans might possibly be attributed at least in part, to the enhanced ^18^F-FDG extravasation in BAT.

With an advantage of high diffusiveness throughout the body, PLPEG-CNTs will provide an effective tool to trace fine fluid kinetics changes in various tissues of a living body.

## Conclusion

Fasting induced disarrangement of collagen bands in the extracellular matrix and widening of the intercellular space in murine BAT. In addition to these, the vascular permeability was enhanced, which was determined by using PLPEG-CNT as a NIR fluorescent probe to trace blood and fluid flow in sub-tissues level. Namely, intravenously administered PLPEG-CNTs accumulated more intensively in BAT in fasted mice than ad lib fed mice, where PLPEG-CNTs extravasated and re-distributed in parenchymal areas as well as non-parenchymal cells. These findings strongly suggest that fasting-induced ^18^F-FDG-PET/CT signal enhancement for human BAT reflects increments in permeability in BAT vessels. Owing to their high diffusivity and the advantage that they emit NIR fluorescence, PLPEG-CNTs have a possibility to be an effective tool to trace fluid flow in sub-tissue levels, which is a challenge as a new application of PLPEG-CNTs in addition to the conventional whole-body vascular imaging.

## Methods

### Preparation of PLPEG-CNT dispersion solution

About 100 mg of CNTs (HiPco, Lot No. R1–831, NanoIntegris, Inc., Skokie, Il, USA) in 100 mL of an aqueous solution of sodium cholate (SC; 1% in weight; Sigma-Aldrich Co. LLC, St. Louis, MO, USA) were homogenized by using an ultrasonic homogenizer (Sonifier 250D, Branson Ultrasonics, Emerson Japan, Ltd. Kanagawa, Japan) at 30% amplitude for for 3 h and centrifuged at 50 000 rpm (210 000 g) for 2 hours with an angle rotor (S50A, Hitachi Koki Co., Ltd. Tokyo, Japan). 80% of the supernatant was collected and centrifuged again at the same condition, and 70% of the resulting supernatant was collected. To concentrate CNTs and exchange the dispersant in the solution, centrifugal filter unit was used (Amicon Ultra-15 Ultra cel-3k, Merck Millipore Corp., Darmshtadt, Germany). To the 10 ml of the supernatant, 2 ml of 1% phospholipid polyethylene glycol aqueous solution (PLPEG; 1,2-distearoyl-sn-glycero-3-phosphoethanolamine-N-[amino(poly(ethylene-glycol))-5000], SUNBRIGHT® DSPE-050PA, NOF American Corporation, New York, USA) was added and concentrated using the filter unit at 2330 g to ~6 ml. 6 ml of water was added to the concentrate and filtered again. This process was repeated 5–7 times. Finally, the solution was concentrated to 2 ml which contained 0.5 mg/ml CNTs and 1% PLPEG (the CNT concentration was estimated from the optical absorbance at 280 nm using a calibration line). The optical absorption spectra and PL spectra were measured with a UV-vis-NIR spectrometer (UV-3600, Shimadzu, Kyoto, Japan) and a spectrofluorometric equipment (NanoLog, HORIBA Ltd., Kyoto, Japan) equipped with a liquid nitrogen-cooled InGaAs array detector, respectively.

### Near-infrared photoluminescence imaging

A home-built imaging system was used for the mouse whole body imaging and paraffin-embedded tissues as described previously^18^. A lamp (IP-307TCS, Lanics; 800-nm short pass filter, FIT Leadintex, Inc., Tokyo, Japan) was placed obliquely above the mouse, and the NIR camera (InGaAs-array video camera, NIRvana 640ST, Princeton Instruments, Trenton, NJ, USA), above the mouse. An objective lens (Cosmicar, Pentax, RICOH Imaging Company, Ltd., Tokyo, Japan) and 1000 nm long-pass filter were used to exclude lights other than CNT fluorescence. The NIR camera exposure time was 100 ms.

### Histological observation

The near infrared fluorescence microscopy was carried out using an epi-illumination-type optical microscope (BX-51 IR, Olympus Corp., Tokyo, Japan) was introduced in detail elsewhere^18^. It was equipped with a Xe lamp (UXL-76XB, Ushio Inc., Tokyo Japan), a single-band bandpass filter (708/75 nm; center: 708 nm, width: 75 nm; BrightLine®, Semrock, IDEX Corp, Lake Forest, IL, USA), and a 785 nm laser single-edge laser-flat dichroic beam splitter (BrightLine®, Semrock, IDEX Corp.). The specimen fluorescence passed through the 785 nm laser single-edge laser-flat dichroic beam splitter, an 808 nm best-value long-pass edge filter (EdgeBasicTM, Semrock, IDEX Corp), and a 1100 nm long-pass filter (Olympus, Tokyo, Japan), before reaching the NIR camera. This equipment also had the halogen lamp and CCD camera (AUSB.3, 4203 K, ARMS system Co., LTD., Tokyo, Japan), by which visible-light transmission images were taken.

For NIR fluorescence microscopy, the formalin-fixed tissues were stained with nuclear fast red (NFR). NIR fluorescent intensity of the NFR reagent was low enough to observe the CNT fluorescence image by 708 nm excitation. To observe collagen bands, the formalin-fixed tissues were stained by silver impregnation.

### Mouse experiments

As similar to the previous report^18^, nude mice (BALB/cAJ1-nu/nu, Female, 7 weeks old; CLEA Japan Inc., Tokyo, Japan) were used. We used the nude mice because hairs of hairy mice diffuse light and make the imaging of mice body difficult. If hairy mice were used, hairs must be removed for imaging, when the skin were damaged and PLPEG-CNTs could accumulate at the damaged-skin site, influencing the biodistribution of PLPEG-CNTs. Before the PLPEG-CNTs injection, the mice were acclimatized to the institute laboratory for 2–3 weeks, and PLPEG-CNT was injected in mice from tail veins. Dose was 0.2 mL/mouse (about 5 mg/kg CNT/body weight). The mice were anaesthetized with isoflurane inhalation solution (Pfizer Inc., New York, USA) for the whole-body imaging with the NIR camera. After the imaging, the mice were euthanized by sampling blood from the vena cava. The adipose tissues and organs were collected, which fixed using formalin or glutaraldehyde.

Mice number used in the whole-body imaging was n = 3–5, and the histology study, n = 5.a.

All the animal experiments were performed in accordance with the regulations approved by the Animal Care and Use Committee of National Institute of Advanced Industrial Science and Technology and Hokkaido University.

### Gene expression analysis of iBAT

Total RNA was extracted using RNAiso (Takara Bio, Shiga, Japan) according to the manufacture’s protocol. The mRNA levels were measured quantitatively by real-time RT-PCR, and normalized using mRNA expression of *ActB* as an internal standard. Briefly, 2 µg of total RNA was reverse transcribed using an oligo (dT) 15-adaptor primer and M-MLV reverse transcriptase (Thermo Fisher Scientific Inc. Waltham, MA). Real-time PCR was performed using a fluorescence thermal cycler (LightCycler system; Roche Diagnostics, Mannheim, Germany), with SYBR Green (Brilliant III Ultra-Fast SYBR Green QPCR Master Mixes, Agilent Technologies, Palo Alto, CA) employed as a double-strand DNA-specific dye, in accordance with the manufacture’s protocol. Primers used are listed in Supporting Data 16.

### PLPEG-CNT/murine sera reactions

PLPEG-CNT (CNT: 0.5 mg/mL, 125 mL) was mixed with 50% diluted murine sera (125 mL) in a 1.5 mL tube, which was kept rotated at 4 °C overnight. The reaction supernatants were subjected to Western blotting studies using a rabbit polyclonal anti-apolipoprotein B antibody (Abcam Plc., Tokyo, Japan, Catalogue No. ab20737), a rabbit polyclonal anti-apolipoprotein E antibody (Abcam Plc., Catalogue No. ab 83115), or a rabbit monoclonal anti-bovine serum albumin antibody (Abcam Plc., Catalogue No. ab192603), along with a horseradish peroxidase-conjugated anti-rabbit IgG secondary antibody (Cell Signaling Technology, Inc., Beverly, MA, USA). In some experiments, Can Get Signal® Immunoreaction Enhancer Solution (TOYOBO Co., Ltd., Catalogue No. NKB-101) was used. Chemiluminescence reactions were performed using ECL Western blotting detection reagents (GE Healthcare UK, Ltd., Buckinghamshire, England) or SuperSignal™ West Dura Extended Duration Substrate (Thermo Fisher Scientific Inc., Tokyo, Japan). Visualisation and intensity measurements of protein bands were performed by development on X-ray films or using a C-DiGit® chemiluminescent Western blot scanner (LI-COR, Inc., Lincoln, NE, USA).

### Transmission electron microscopy and staining

The detailed methods were described elsewhere^18^. Briefly, the tissue blocks with sizes of 1–2 mm were fixed with glutaraldehyde, stained with an aqueous solution containing 1% OsO_4_, embedded in in epoxy resin (Epon812), and sliced (thickness 80 nm) for the TEM observations. These observations were performed with an acceleration voltage of 80 kV (H7600 TEM, Hitachi, Ltd, Tokyo, Japan).

## Electronic supplementary material

Supplementary Information

## Data Availability

The datasets generated during and/or analysed during the current study are available from the corresponding author on reasonable request.
