# Supplementary material for: Fasting-dependent Vascular Permeability Enhancement in Brown Adipose Tissues Evidenced by Using Carbon Nanotubes as Fluorescent Probes
Source: Sci Rep. 2018 Sep 27;8:14446. doi: 10.1038/s41598-018-32758-8 (PMC6160465; doi:10.1038/s41598-018-32758-8)
Supplement: Supplementary file 1 — Supplementary Information [file 41598_2018_32758_MOESM1_ESM.pdf]

Supporting Data

Fasting-dependent Vascular Permeability Enhancement in Brown Adipose Tissues Evidenced by Using Carbon Nanotubes as Fluorescent Probes

Masako Yudasaka[1,2]*, Yohei Yomogida[1], Minfang Zhang[3], Masako Nakahara[4], Norihiko Kobayashi[4], Takeshi Tanaka[1], Yuko Okamatsu-Ogura[5], Kumiko Saeki[4]*, Hiromichi Kataura[1]*

E-mail: m-yudasaka@aist.go.jp, h-kataura@aist.go.jp, saeki@ri.ncgm.go.jp

Keywords: fasting, brown adipose tissue, extravasation, carbon nanotube, near infrared fluorescence microscopy e

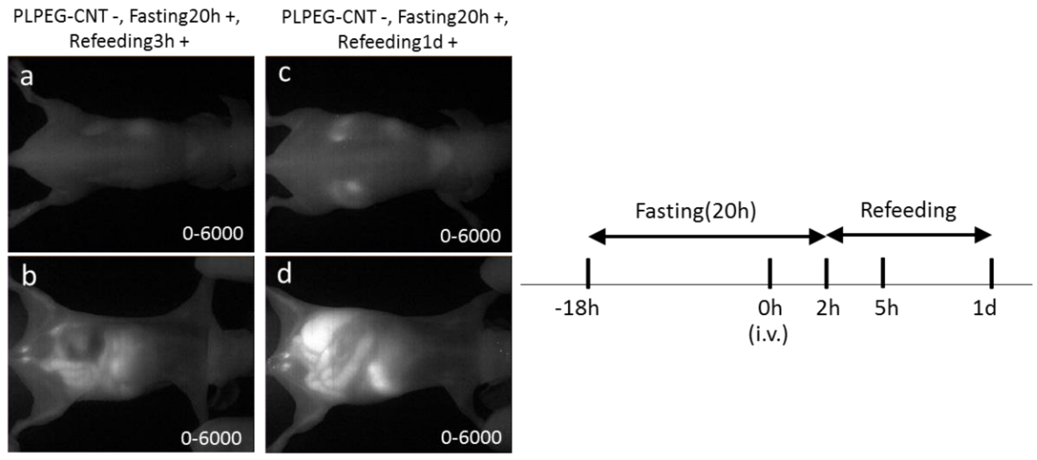

Supporting Data 1. NIR photographs of fasted mice without injection of PLPEG-CNT. Post injection time (PIT) was 5h for “a, b” or 1d for “c, d”. The time schedule is presented below the images. Image intensity range was “0-6000”. The vasculatures were were not visible without PLEG-CNT injection. The gastrointestinal system created bright images due to auto-fluorescence of refeeding-food ingredients. (n=4)

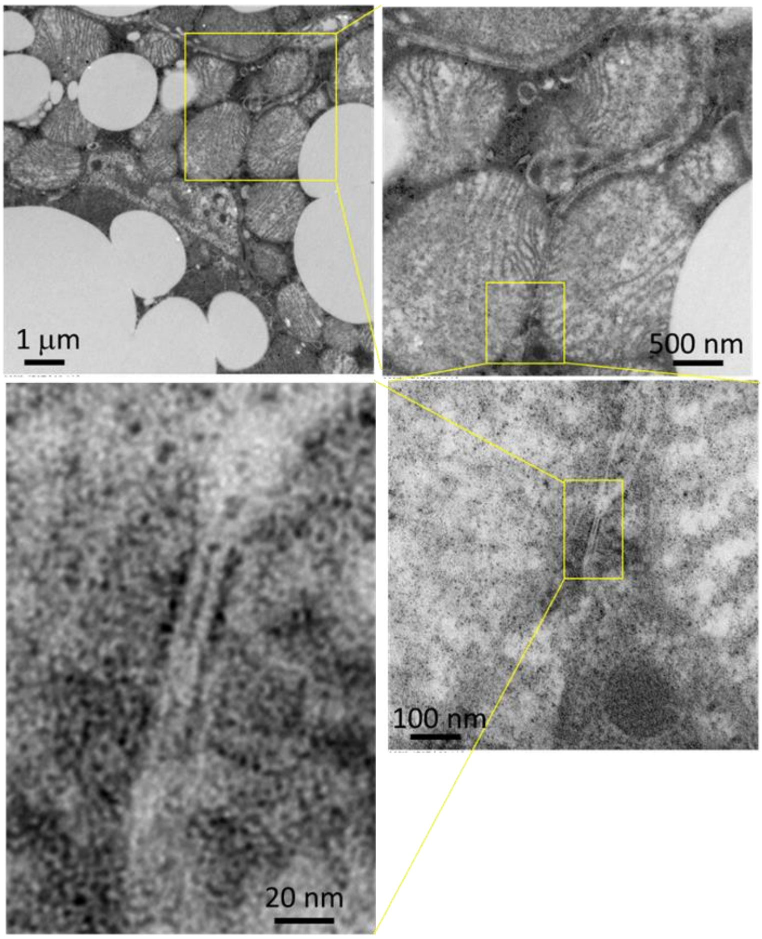

Supporting Data 2. TEM images of iBAT of a control mouse (PLPEG-CNT -, Fasting -). Gap junction was observed in left-bottom panel, showing the gap junction width of about 20 nm. (n=1)

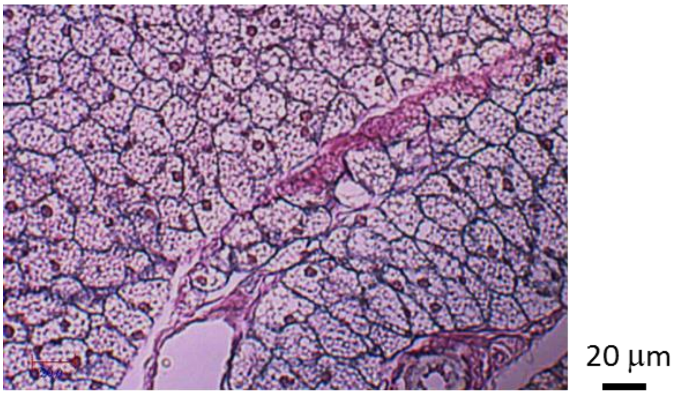

Supporting Data 3. Optical micrographs of iBAT of a mouse (PLPEG-CNT -, fasting18h +, Refeeding 1d +) after refeeding for 1 day. Tissue was stained with silver-impregnation. (n=5)

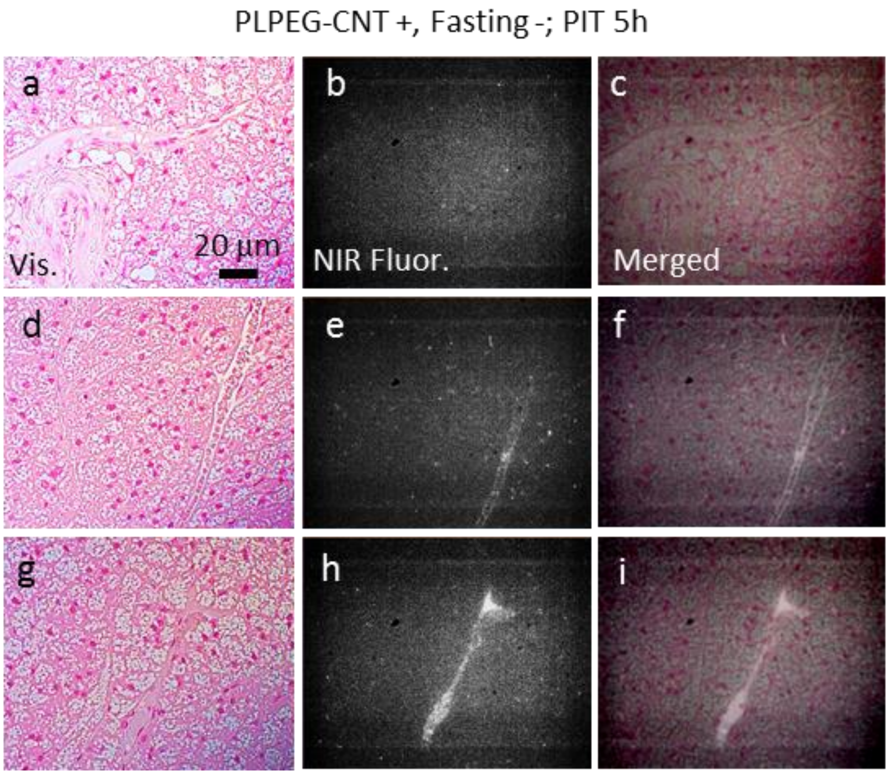

Supporting Data 4. Optical micrographs of iBATs of non-fasted mice (PLPEG-CNT +, Fasting -, PIT 5h). CNT-fluorescence spots in the parenchyma area were weak in brightness and small in size. Those in the vessels were localized clearly (“d-f”, “g-i”). Staining: Nuclear fast red. (n=5)

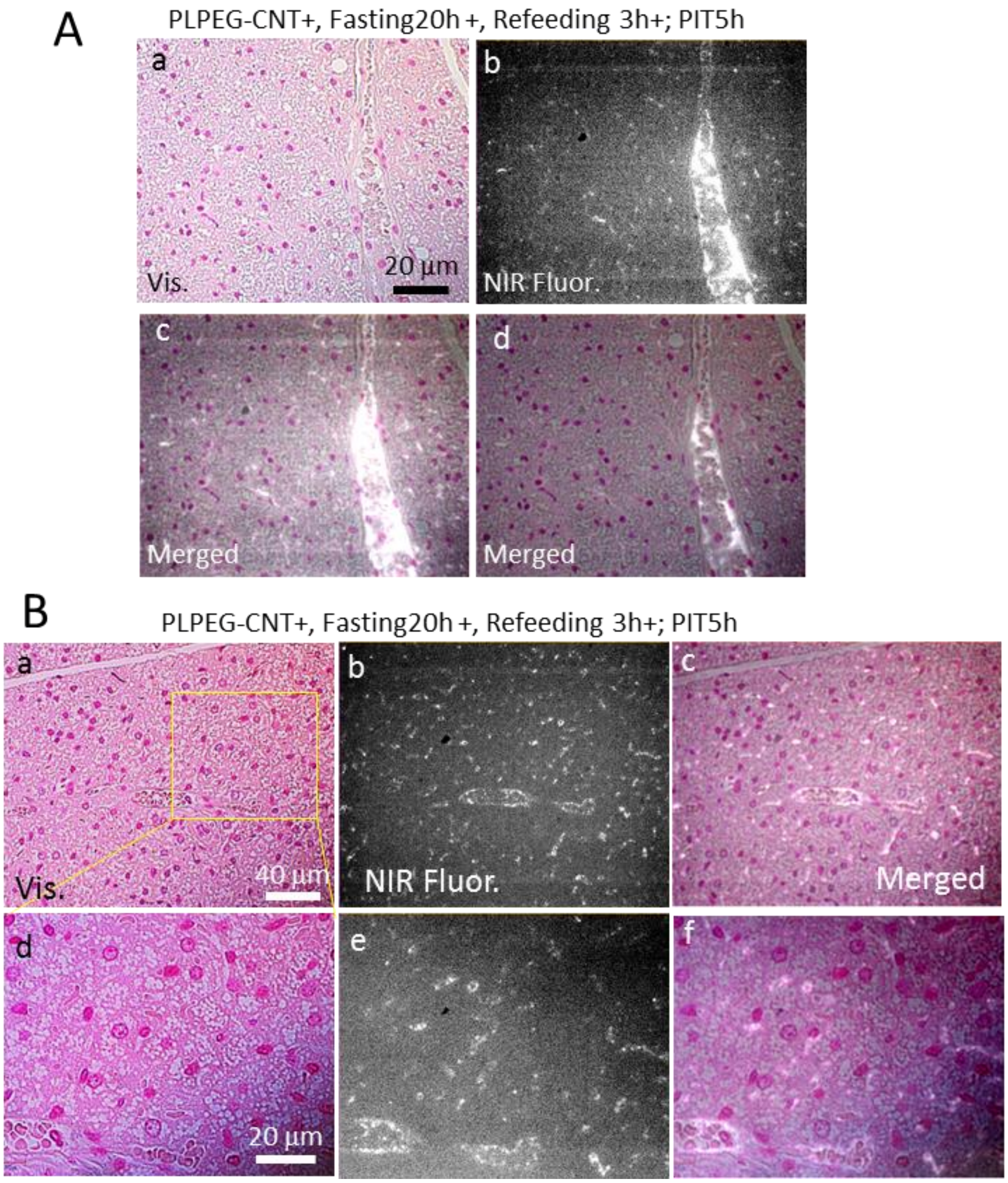

Supporting Data 5. Optical micrographs of aBATs of fasted mice. CNT-fluorescence spots were localized in the vessels and parenchyma area (A, B). In the parenchyma area, CNT-fluorescence spots were found near the nuclei of BA cells or in the inter-cellular areas (B). The brightness and contrast of images were adjusted for easy visualization of CNT-fluorescence spots in the parenchyma area in "A-c", and in the arteriole in "A-d". (n=5)

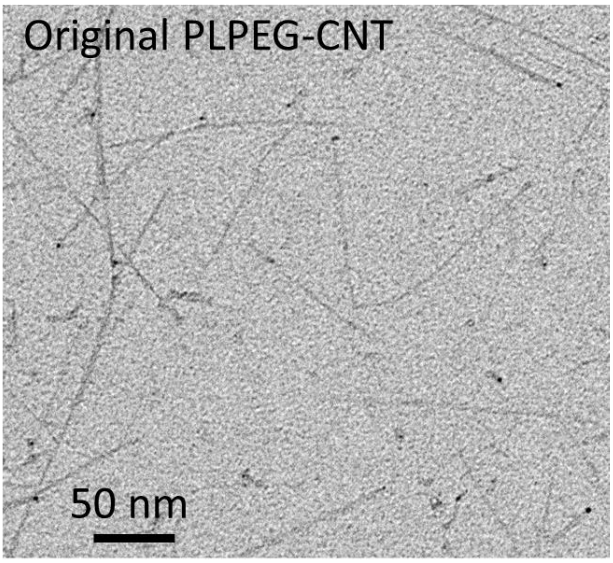

Supporting Data 6. TEM image of original PLPEG-CNTs. Dark-appeared particles indicated the iron catalysts used for the CNT production.

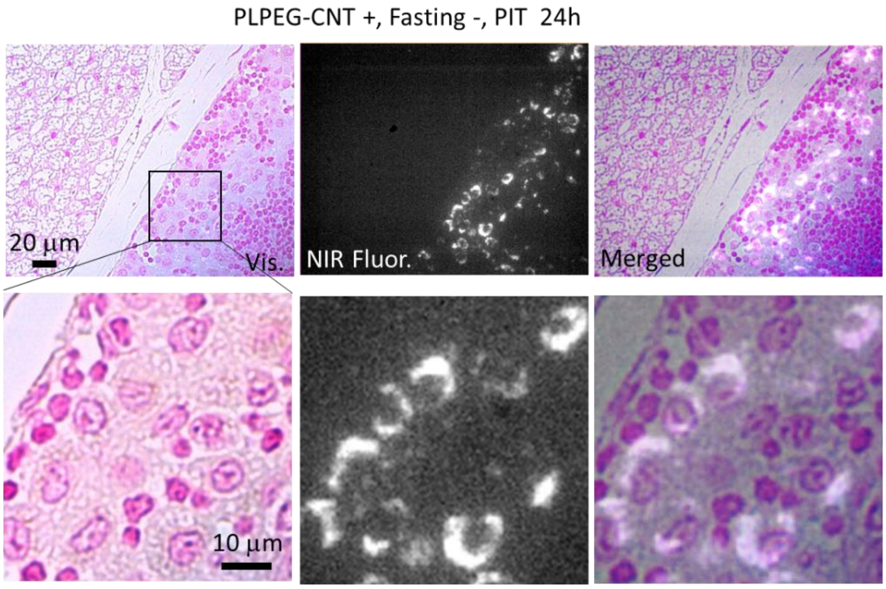

Supporting Data 7. Optical micrographs of lymph nodes in aBAT of a mouse (PLPEG-CNT +, Fasting -; PIT 24h). Bright spots in NIR fluorescence and merged images correspond to CNTs fluorescence. The CNT-fluorescence bright spots often existed near the nuclei of reticular cells. (n=5)

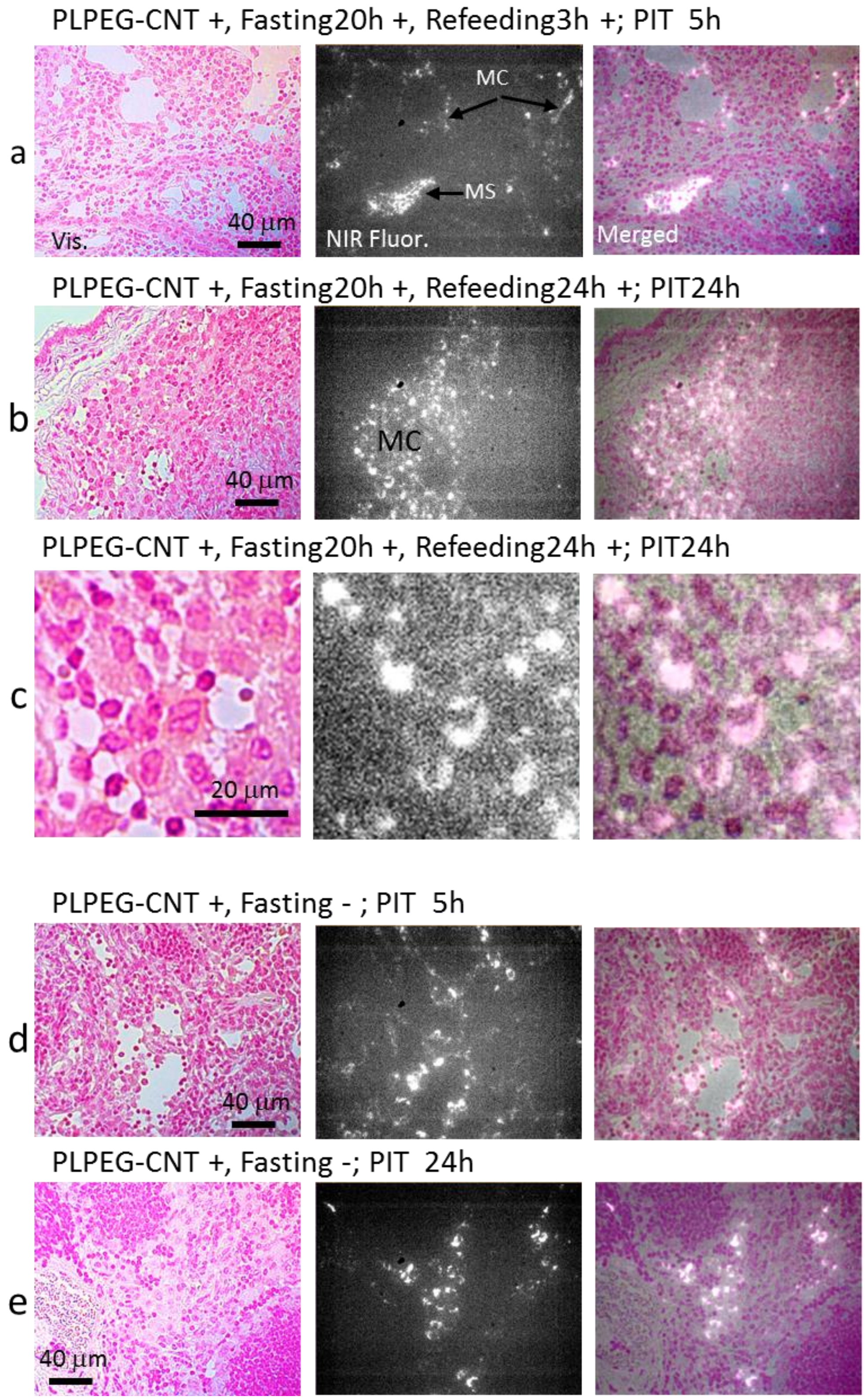

Supporting Data 8. Optical micrographs of lymph nodes in ingWATs of mice. CNT-fluorescence spots were localized in medullary cords and medullary sinus, which did

not depend on the fasting or the post injection time (a-e). The CNT-fluorescence bright spots often existed near the nuclei of reticular cells (c) as similar in the case of lymph node in aBAT. Bright spots in NIR fluorescence and merged images correspond to CNTs fluorescence. Staining: Nuclear fast red. MC: medullary cord, MS: medullary sinus. (n=5)

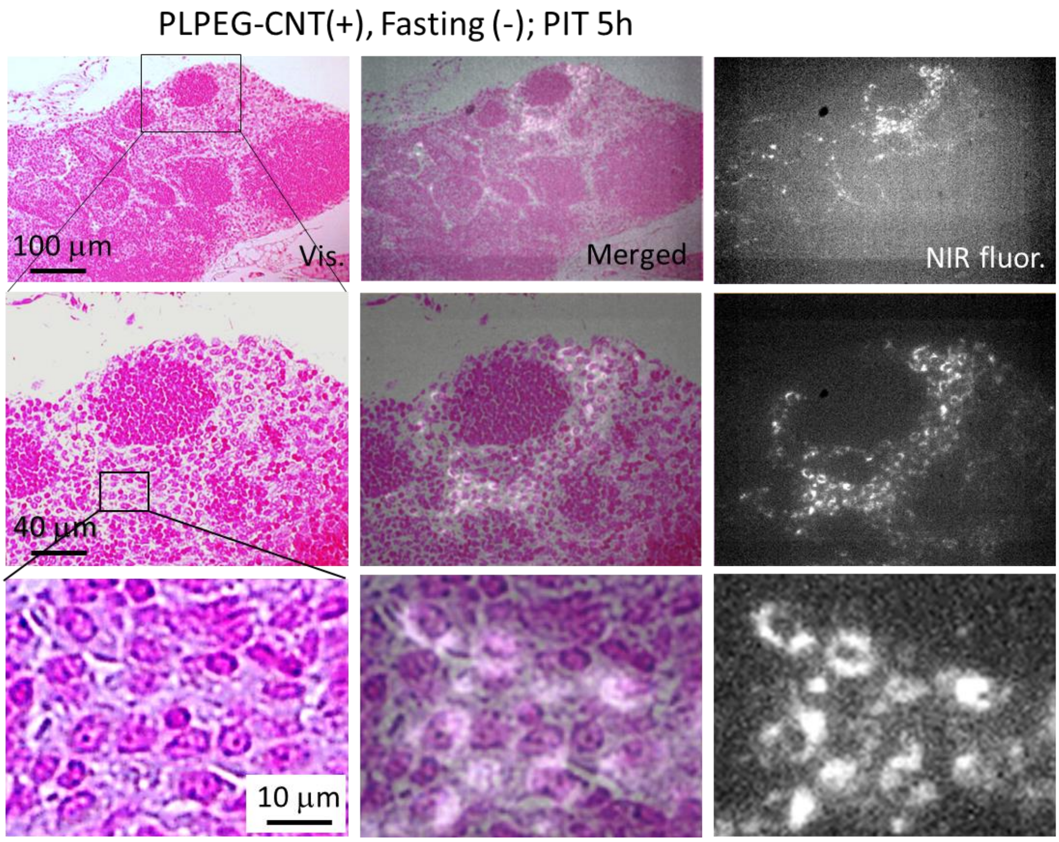

Supporting Data 9. Optical micrographs of mandibular lymph nodes of PLPEG-CNT injected and fasted mice at PIT 5h. Bright spots in NIR images correspond to the CNTs fluorescence. CNTs were found in the reticular cells mainly in medullary cords. Staining: Nuclear fast red. (n=5)

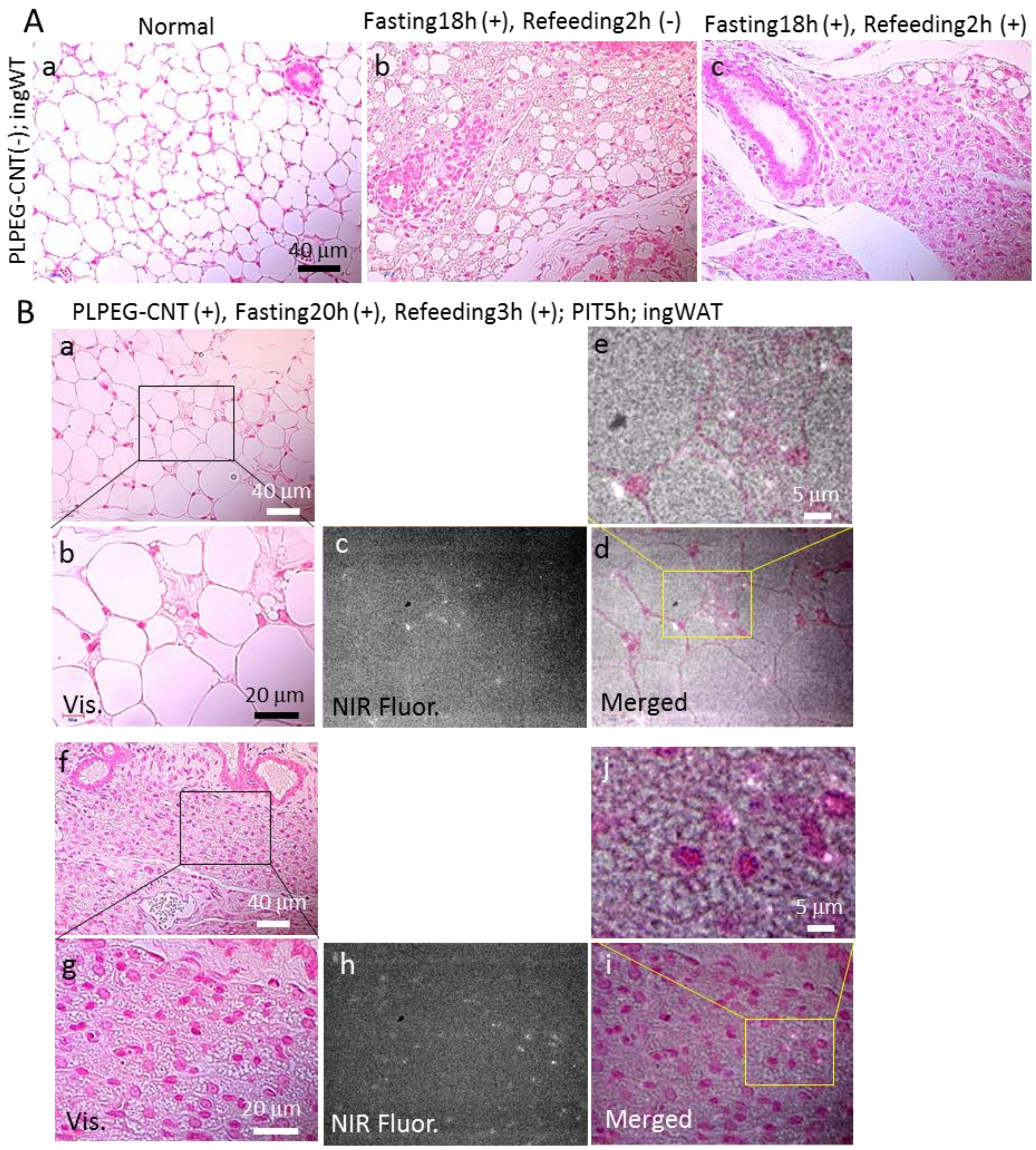

Supporting Data 11. Optical micrographs of ingWATs of mice without PLPEG-CNT injection (A): Normal mouse (A-a), mouse (Fasting18h +, Refeeding -) (A-b), and mouse (Fasting18h +, Refeeding2h +) (A-c). Optical micrographs of ingWAT of PLPEG-CNT injected mouse (PLPEE-CNT +, Fasting20h +, Refeeding3h +; PIT5h) (B). Images for WA-cell area (B a-e) and high cell-density area (B f-j). Staining: Nuclear fast red. (n=5)

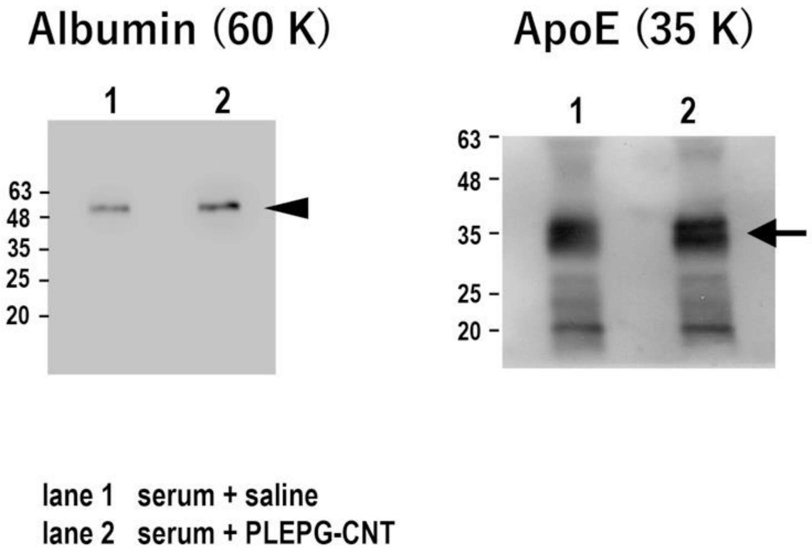

Supporting Data 12. Serum absorption assay. Murine serum was mixed with either saline (lane 1) or PLPEG-CNT/saline (lane 2) and incubated as previously reported*. The supernatant of the reaction mixture was subjected to Western blotting using an anti-albumin antibody (left) or anti-ApoE antibody (right).

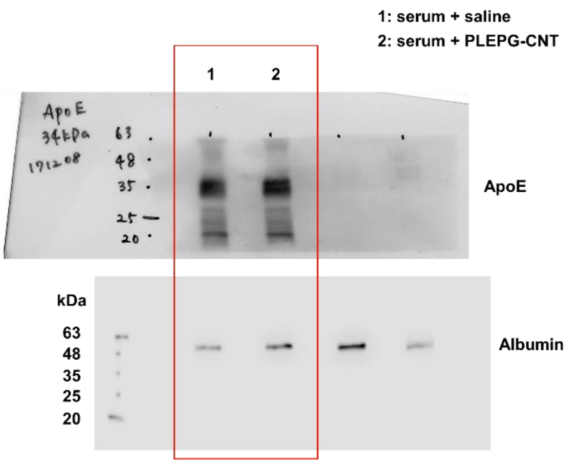

Supporting Data 12-1. Supplementary full length Western blot gel.

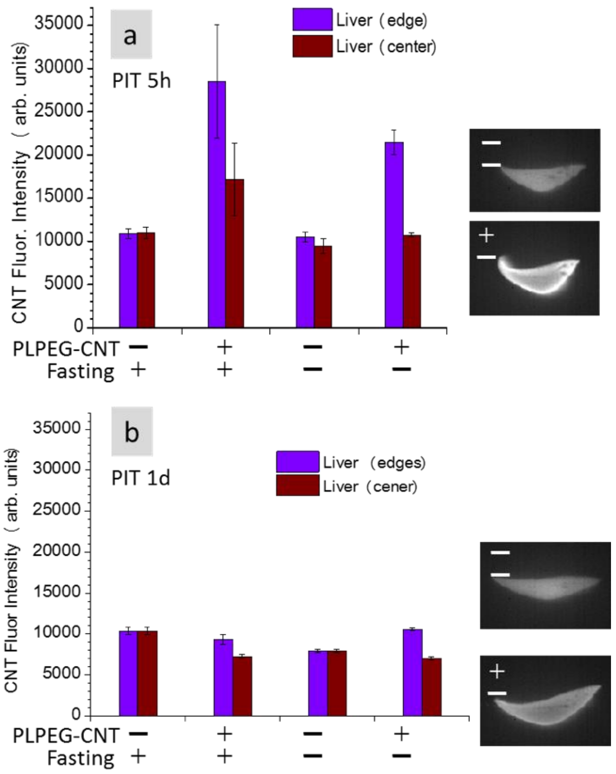

Supporting Data 13. CNT fluorescence intensities of liver tissues embedded in paraffin blocks. Post injection times are 5h (a) and 24h (b). BG 1500 was subtracted in (a) and (b). Insets are the NIR fluorescence micrographs of livers embedded in paraffin blocks. At PIT 5h, PLPEG-CNT injected mice presented bright liver-edges, but not for mice without PLPEG-CNT injection. The CNT-fluorescence became dark after 24h, which reason is not clear but PLPEG-CNT excretion via virial routes may partly contribute. (n=5)

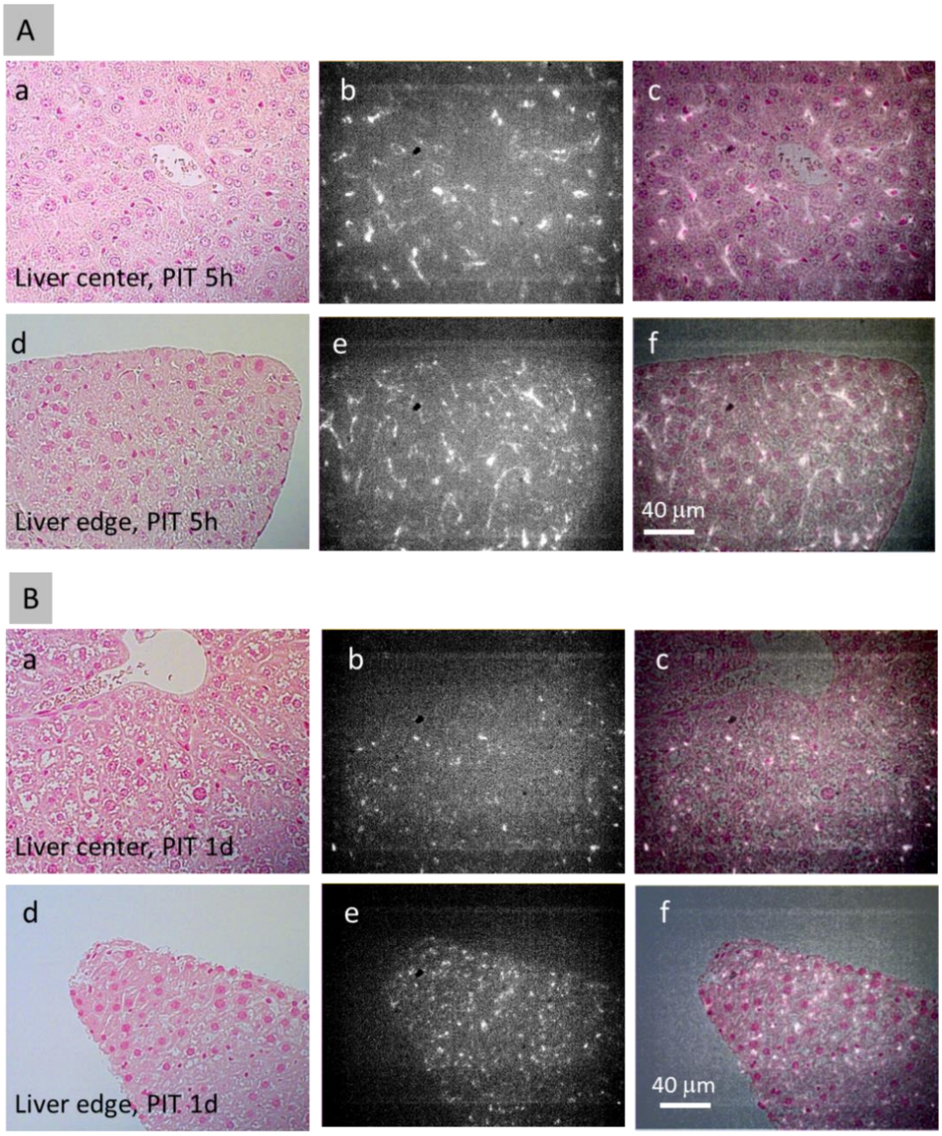

Supporting Data 14. Optical microscopy observation of liver tissues in mice (PLPEG-CNT +, Fasting+) at PIT 5h (A) and PIT 1d (B). The micrographs at the center area (a-c) and edge area (d-f). (n=5)

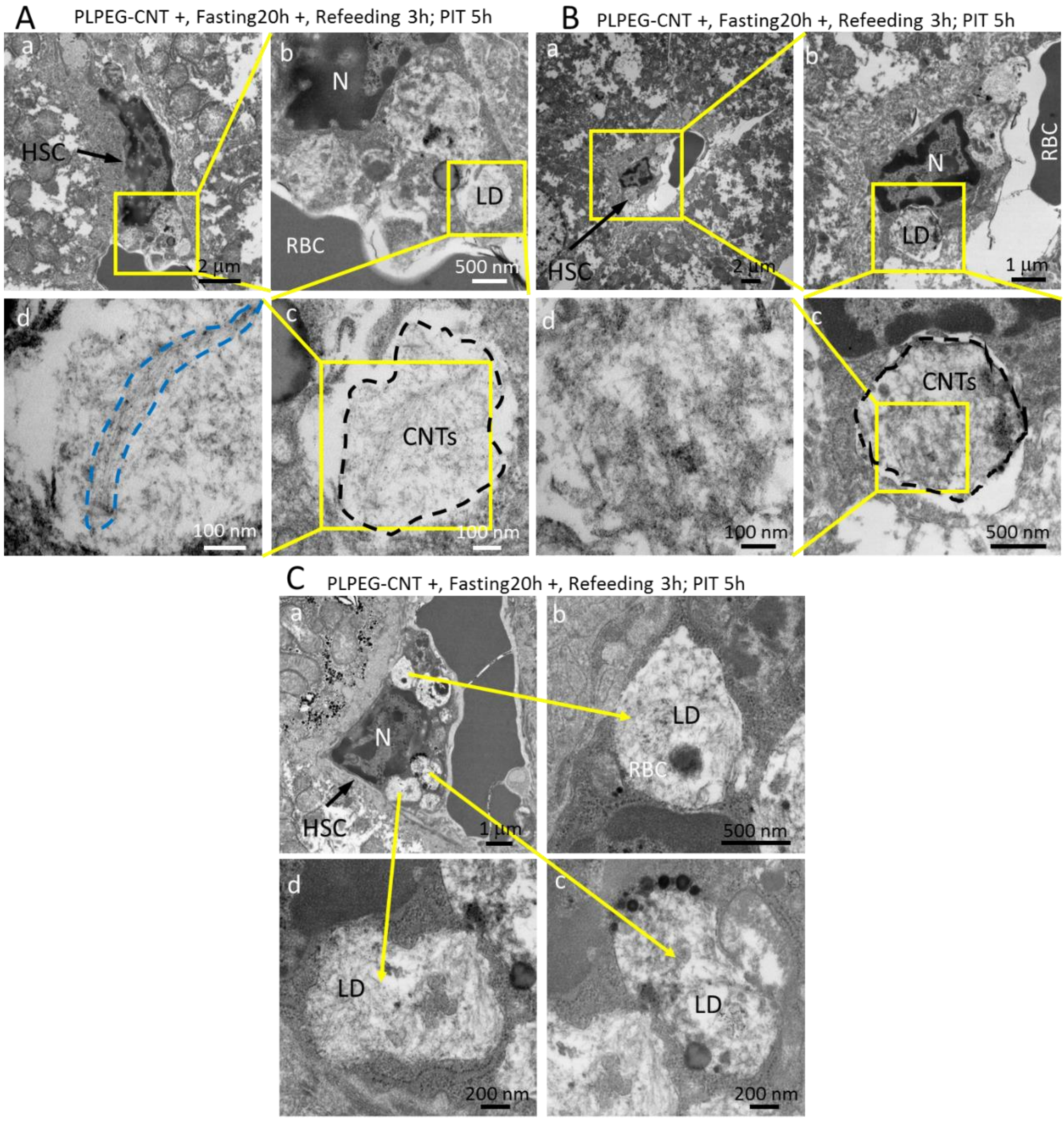

Supporting Data 15. TEM images of liver in the mouse (PLPEG-CNT +, Fasting20h +, Refeeding 3h; PIT 5h). Hepatic stellate cells (HSCs) are indicated with black arrows (A-C). CNTs inside lipid droplets (LDs) of hepatic stellate cells are encircled with black brokwn lines (A, B). Yellow arrows indicate lipid droplets containing CNTs in hepatic stellate cells (C). Fibrous CNTs are densely accmulated in the LDs, and they sometimes aligned parallely as seen in an area enclosed with a blue broken line in A-d. RBC: Red blood cell, N: Nucleus. (n=1)

Supporting Data 16.

Table1 Primers used in Real-time PCR

Ucp1	Forward	5’ - GTG AAG GTC AGA ATG CAA GC - 3’
	Reverse	5’ - AGG GCC CCC TTC ATG AGG TC - 3’
Col3a1	Forward	5’ -AAG CAC TGG TGG ACA GAT TC - 3’
	Reverse	5’ -TCT TCA GGA AGA TCA GGA GG - 3’
Cx43	Forward	5’ - ACT TCA GCC TCC AAG GAG TT- 3’
	Reverse	5’ - AAA TGA AGA GCA CCG ACA GC- 3’
Atg5	Forward	5’ - AAA GAC CAC AAG CAG CTC TG- 3’
	Reverse	5’ - CGG AAC AGC TTC TGG ATG AA- 3’
Lc3b	Forward	5’ - TCC GAG AAG ACC TTC AAG CA - 3’
	Reverse	5’ - ACC AGG AAC TTG GTC TTG TC- 3’
Vegfa	Forward	5’ - TGA ACT TTC TGC TCT CTT GGG T- 3’
	Reverse	5’ - ACT TGA TCA CTT CAT GGG ACT TC- 3’
Vegfb	Forward	5’ - AAC ACC AAG TCC GAA TGC AG- 3’
	Reverse	5’ - CTG TCT GGC TTC ACA GCA CT - 3’
Mmp3	Forward	5’ - TCA CCA ATG TGC AGC TCT AC - 3’
	Reverse	5’ - CCT CAT ATG CAG CAT CCA TG- 3’
Mmp14	Forward	5’ - GAT GGA TGG ATA CCC AAT GC - 3’
	Reverse	5’ - CGT CAA ACA CCC AGT GCT TA - 3’
ActB	Forward	5’ - TCG TTAC CAC AGG CAT TGT GAT - 3’
	Reverse	5’ - TGC TCG AAG TCT AGA GCA AC - 3’
